# New feeding mechanism to enhance the properties of wrapped elastic composite yarn and denim fabrics thereof

**DOI:** 10.1016/j.heliyon.2024.e35803

**Published:** 2024-08-05

**Authors:** Osman Babaarslan, Esin Sarıoğlu, Onur Duru

**Affiliations:** a Textile Engineering Department, Çukurova University, Adana, Turkey; b Department of Textile and Fashion Design, Gaziantep University, Gaziantep, Turkey; c Research&Development Center, Bossa Ticaret Ve Sanayi İşletmeleri T.A.Ş, Adana, Turkey

**Keywords:** Dual-core spun yarn, Wrapped elastic composite yarn, Denim fabric, New feeding mechanism, Modified ring spinning system, Performance

## Abstract

In light of the recent technological advancements in composite yarns with multicomponent cores, also known as dual core spun yarns, their utilisation in the textile industry has become widespread. A V-grooved roller is employed to enhance the feeding of multicomponent cores. In this context, diverse composite yarn designs with varying feeding mechanisms can be employed to optimise yarn performance. This study presents an alternative feeding mechanism utilising a W-grooved roller for commercial use in composite yarn production. To achieve this, dual core yarns and wound elastic composite yarns (filament feeding varies with the sheath cotton fibre with elastane in the centre wrapped on the right and left side) were produced using a modified ring spinning system with different parameters. Denim fabrics were then produced using these composite yarns as weft yarns. The results demonstrated that the wrapped elastic composite yarns with left and right filament core positioning exhibited superior yarn properties in terms of strength, elongation at break, evenness and hairiness. Furthermore, denim fabrics made with wrapped elastic composite yarns exhibited superior breaking load and tearing force results on weft basis. These fabrics also exhibited lower growth, defined as the percentage of the fabric returning to its original length after tensile stress removal.

## Highlights

• An alternative apparatus of W-grooved roller to enhance the wrapped multicomponent core spun yarn instead of V-grooved roller feeding apparatus for dual core spun yarn.
• Right and left sided feeding of filament can be achieved by W-grooved roller.
• Improving composite yarn properties compared with dual core spun yarn.
• W-grooved roller is a patented apparatus that can be adapted easily to the ring spinning machine.

## 1 Introduction

Core-spun yarns are traditionally produced on ring spinning machines by adding elastane to the centre of the yarn using a V-grooved cylinder positioned behind the front rollers of the drafting unit. Different combinations of filaments (elastane and semi-elastic filaments) and staple fibres can be used to create various core-spun yarn structures on a conventional ring frame with modifications [[1], [2], [3], [4], [5], [6], [7], [8], [9], [10], [11], [12], [13], [14], [15]]. In what can be considered a pioneering study, Sawhney et al. developed a mechanical staple core-spun spinning device to produce polyester core-spun cotton winding yarns on a modified ring spinning machine [1]. In another study, Sawhney et al. conducted further research on the system proposed in their previous study, investigating the production of cotton winding filament core yarn. In their study, the researchers obtained filament core yarns using two cotton rovings, which represents a novel technique for comparison with conventional yarns. The results demonstrated that the cored yarns produced using the proposed new technique exhibited excellent core coverage and satisfactory sliver resistance [2]. Pourahmad and Johari employed a modified ring spinning machine to eliminate the slippage of staple sheath fibres relative to the core, a problem encountered in core-spun yarns and known as the barberpole effect. This was achieved by using three strands of sheath fibres and three core filaments [7]. Gharahaghaji et al. proposed a novel approach to yarn production, termed cluster spun yarn, which involves the integration of cotton fibres and polyester multifilaments within a slottedd roller within a modified ring spinning system, with a view to comparing its properties with those of conventional core-spun yarn [8]. Naeem et al. devised a novel technique involving a micro-grooved roller and multifilament guide mounted on a conventional ring spinning machine, with the objective of producing staple core filament wound composite yarn [10]. Matsumoto et al. investigated an innovative method for creating a new type of composite yarn known as twin staple-core spun yarn. This technique involved a single twisting process, resulting in a yarn with a unique three-layer structure consisting of two adjacent core layers made of staple fibre and an outer sheath layer. The authors emphasised that the success of this method depends on the careful management of spinning tensions between the sheath and core layers, the adjustment of twist levels, distances between rovings and the correct selection of fibre types and lengths [12].

Significant progress has been made in the production of multicomponent composite yarns, commonly referred to as dual core yarn. This advanced textile yarn contains two distinct filaments at its core, frequently accompanied by a sheath of staple fibres, such as cotton. The unique structure of this yarn enhances the strength, elongation and flexibility of the resulting fabric, particularly when subjected to heat treatments such as dyeing and finishing. It offers enhanced durability while maintaining flexibility, rendering it an ideal choice for stretchy and comfortable garments, including jeans and sportswear. It is evident that research has been conducted on the utilisation of various raw materials in the production of dual core spun yarn. In accordance with the intended use, multicomponent yarn production has been achieved in a conventional ring spinning system utilising a combination of filaments, including PBT/spandex, CM800/spandex, T400/spandex, SPH/spandex, tungsten/elastane, copper/elastane, polyester/elastane, microfilament polyester/elastane, and PTT/elastane [[16], [17], [18], [19], [20], [21], [22], [23], [24], [25]]. In these studies, it was observed that not only was the base material of the yarn distinct, but also that the effects of sheath fibres with varying properties on yarn and fabric properties were investigated. Furthermore, research has focused on the characteristics of sheath fibres, including wool, polyester, cotton, polyester/cotton blends, viscose, lyocell/cotton blends, hemp, organic cotton etc. Studies have explored the production of dual core spun yarns using different raw materials and blend ratios, as well as determining the fabric performance characteristics [[26], [27], [28], [29], [30], [31]].

A number of studies have examined the performance characteristics of yarn and fabric when more than two components are added to the yarn core [32,33]. Additionally, research has been conducted on the use of functional filaments at the core of the yarn to provide durable antibacterial and temperature-regulated properties [34]. Dual core-spun yarns are commonly used in denim fabric production. Although these yarns are typically employed as weft yarns in the production of denim fabric, there are studies that examine the effects of variable parameters, including the elastane or filament draft/ratio, yarn linear density, and the raw material itself, on the performance of denim fabric. These studies include investigations into the effects of varying parameters on the performance of denim fabric, including the incorporation of elastane or filament draft/ratio, yarn linear density, and the raw material itself [[35], [36], [37], [38], [39], [40], [41], [42], [43], [44], [45]]. One such study, conducted by Ute, focused on improving comfort during body movements by incorporating 10–35 % elasticity in denim jeans. In order to achieve this objective, alternative materials such as bi-component polyester fibres and polybutylene terephthalate were considered as potential core components due to their strength and recovery properties [37]. Erbil et al. investigated the performance of denim fabrics in terms of stretch and recovery, strength, and other important fabric properties when using different yarn types (dual and single core spun yarns) [41]. Rahim et al. provided a detailed account of the production process on an industrial scale, analysing structural parameters, tensile properties, and the elastic recovery behaviour of the yarns under cyclic loading. The findings of the study indicated that the optimal combination of elastane/T400 can yield a yarn with excellent tenacity, elongation, and lower instances of bagging, ultimately contributing to the production of durable stretch jeans that offer high comfort and long-lasting shape retention [42]. Irfan et al. focused on the research of dual-core yarns, which are particularly used in denim manufacturing. The study examined the influence of different filaments (T400, polyester, and polyester textured) and yarn structures (siro and non-siro) on the tensile, elastic, and other properties of dual-core yarns at the same twist level and linear density [43].

This study examines a novel filament feeding mechanism, designated the "W-grooved" roller, which enables feeding from both sides of the yarn. This mechanism is employed in the production of wrapped elastic composite yarns and dual core-spun yarns, using a "V-grooved" roller. Additionally, the study investigates the influence of core type and filament positioning on yarn and denim fabric properties.

## 2 Materials and methods

### 2.1 Materials

This study examined the quality parameters of dual core-spun and wrapped elastic composite yarns. These yarns exhibited diverse core types and distinct positioning of the filament as the second core component. In order to ascertain the effect of the second core components on the quality of the yarn, a number of alternative materials were trialled. These included polyethylene terephthalate (PET) and polytrimethylene terephthalate (PTT) bicomponent commercial elastomer multiester T400 and polybutylene terephthalate (PBT) filaments. Table 1 here provides the properties of these filaments.

**Table 1:** Experimental design.

Parameters	Level 1	Level 2	Level 3
Core Type	T400-Elastane	PBT-Elastane	–
Positioning of Second Core	Left	Right	Center

The first core component utilised was elastane with a linear density of 78 dtex. This was fed in the centre of both the dual core-spun yarns and wrapped elastic composite yarns. For the sheath fibre, cotton fibre with a micronaire of 3.74, a strength of 24.6 g/tex, and an elongation at break of 9.3 % was chosen. The experimental design of this study is shown in Table 2.

**Table 2:** Properties of filament core components of composite yarn.

Properties	T400	PBT
Linear density (dtex)	55	55
Number of filament	34	24
Tenacity (cN/dtex)	3.53	2.10
Breaking elongation (%)	12	4–10

## 3 Methods

### 3.1 The design of wrapped elastic composite yarn

**Fig. 1:**
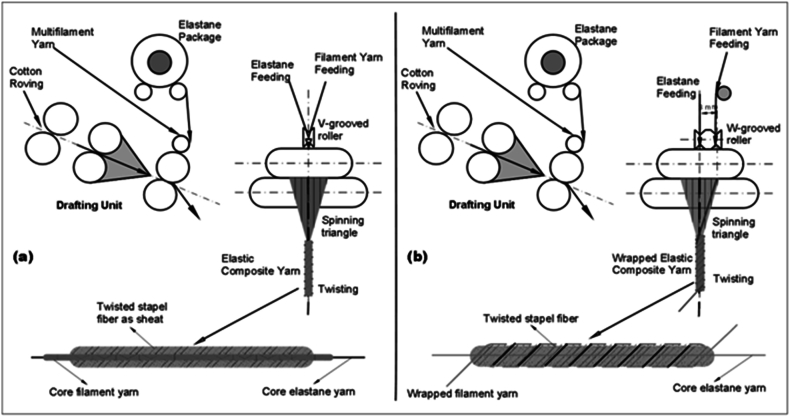
Composite yarn spinning principle; (a) elastane and filament together feeding as a core (dual core-spun yarn), (b) elastane as a core and filament as a wrap feeding (wrapped elastic composite yarn).

Dual core-spun yarn structure includes elastane and filament, which are fed together by means of V-grooved rollers and covered by a staple sheath fiber (Fig. 1(a)).

In the case of dual core-spun yarns, where elastic and non-elastic filament yarns are used to form the composite structure, the tensile strength values decrease. This is due to the fibrous sheath twisting and bending as it moves towards the centre, which reduces the tensile strength. The elastic and non-elastic filaments in the core of the yarn create spaces within the fibrous sheath, reducing the contact surface and preventing sufficient winding. This results in a reduction in tensile strength, which can lead to tearing and breaking in fabrics made from this yarn [46]. Accordingly, enhancements are required in this technical domain to address the aforementioned issues. A number of studies have been conducted with the objective of improving yarn quality and creating diverse yarn structures. One such study is focused on siro wrapped yarn, also known as sirofil yarn, which aims to enhance yarn quality in terms of evenness and tenacity [47]. Sirofil spun yarn is produced using modified siro spinning technology, where a filament replaces one of the siro elements [48]. The yarn is composed of roving and filament components. During the twisting process, the staple yarn is twisted first, followed by the winding of a filament from the right side, left side, or both sides of the staple bundle onto the formed staple yarn [49]. Experimental studies have demonstrated that sirofil yarn exhibits enhanced evenness, reduced hairiness, and increased breaking strength and elongation [14]. Another study examined the design of siro twin core spun yarn, where two identical staple rovings are fed simultaneously to the drafting system, resulting in four types of twin-core spun yarns [18]. These studies exemplified research on the production of twisted composite yarn. In light of the existing literature, a new apparatus, designated the W-grooved roller, has been developed for the production of wrapped elastic composite yarns. This apparatus, depicted in Fig. 1(b), allows for the production of these yarns in a modified ring spinning system. In contrast to the "V-grooved roller," the “W-grooved roller” feeds elastane into one groove while the filament is fed into another groove. Throughout the production process, the elastane in the center and the filament do not come into contact with each other, remaining separate within the composite yarn structure.

**Fig. 2:**
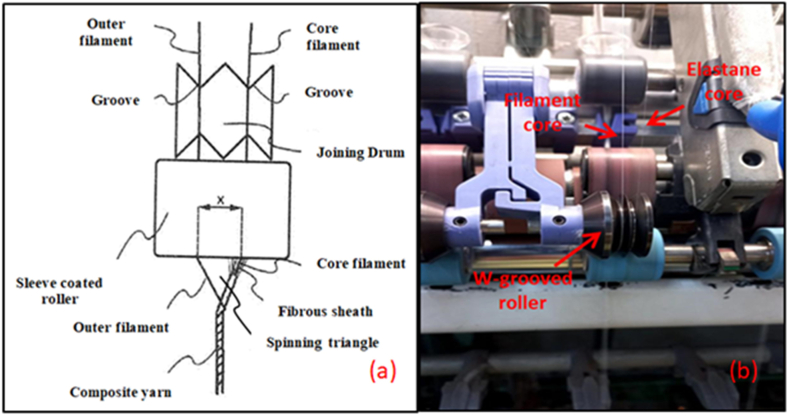
Representation of W-grooved roller feeding mechanism (a) detailed view of the system [45], (b) wrapped elastic composite yarn production photos of modified ring spinning system; left side positioning of filament with center positioning of elastane.

The fibrous sheath is fed to the spinning unit, where the elastane is fed into the center. At the spinning triangle formed at the output of the sleeve-coated roller, the fibrous sheath fully covers the elastane in the middle region. The filament is then twisted together with the fibrous sheath, contributing directly to the composite yarn structure. Fig. 2(a) shows the distance (x) between the filaments that is measured from the point where the elastane passes through the joining drum to the point where the core filament emerges under the sleeve-covered cylinder. This distance (x) can range from 0.1 to 30 mm, preferably between 1 mm and 20 mm [46].

In Fig. 2(b), a photographic view of the "W-grooved" roller feeding mechanism is shown, with the second core filament positioned on the left side and the elastane core positioned in the center.

### 3.2 Composite yarns production

Dual core-spun and wrapped elastic composite yarns with a yarn count of Ne 16/1 were manufactured using a modified ring spinning system. All production parameters were kept constant, including a roving count of Ne 0.7, an elastane draft ratio of 3.62, a filament draft ratio of 1.08, a spindle speed of 12,160 rpm, a twist of 760 turns/m, and a "Z" twist direction.

### 3.3 Denim fabric production

All of the yarn samples produced were used as weft yarn in the production of twill denim fabrics with a 3/1 pattern, using the same production parameters. For this purpose, Ne 14/1100 % cotton ring spun yarn was used as the warp yarn. The denim fabrics were produced using a Picanol brand rapier weaving machine, with the following parameters: warp density of 28 ends/cm, weft density of 20 picks/cm, dent number of 70/4, and machine speed of 550 rev/min. After the production of the denim fabric, singeing, desizing, finishing, and thermal fixation processes were carried out in that order.

### 3.4 Yarn and denim fabric experiments

The produced yarn and denim fabric samples were conditioned at standard atmosphere conditions, with a temperature of 21 ± 1 °C and a relative humidity of 65 ± 2 %, for 24 h before testing. The testing procedures followed the ASTM D1776-08 standard. To determine the tenacity and breaking elongation values of the yarn samples, the Uster Tensorapid 4 was used, following the ASTM D2256 standard. A total of 10 samples were tested for each yarn type, with a load cell capacity of 500 N. The unevenness and hairiness properties of the yarn samples were tested using the Uster Tester-5 device, in accordance with the ASTM D1425 test standard. For the denim fabric samples, the tensile properties were tested according to the ASTM D5034-09 standards. The dynamic tear force was determined using the ElmaTear test device, following the ASTM D1424-09 standard. Additionally, the elasticity and growth analysis of the denim fabrics in the weft direction were performed by applying a 1.35 kg dead load to the specimen, in accordance with the ASTM D3107 standard. The growth values were determined after 30 min and 2 h of relaxation time.

### 3.5 Statistical evaluation

The test results were statistically evaluated using the Minitab® package program. An analysis of variance test was conducted with a 95 % confidence interval. Tukey's multiple comparison test was also performed to assess differences in composite yarn and denim fabric properties among subgroups based on the positioning of the second core parameter.

## 4 Results and discussion

### 4.1 Composite yarn properties

**Fig. 3:**
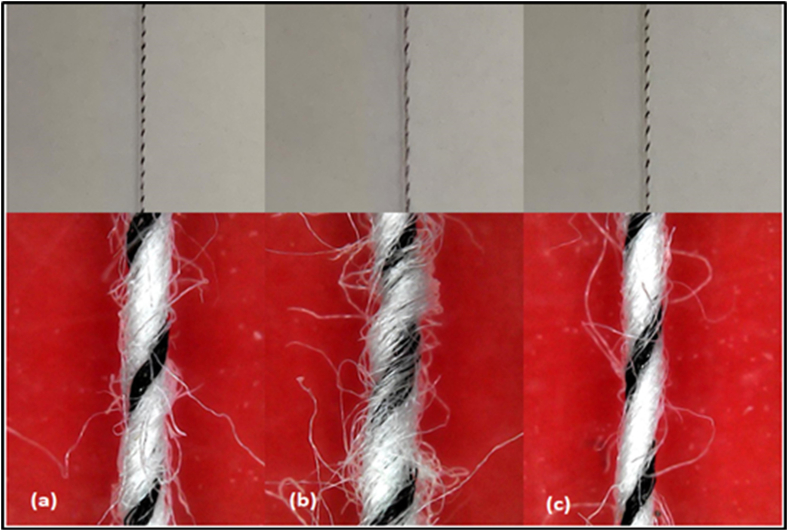
Longitudinal view of composite yarns (a) left side positioning of filament (b) center positioning of filament (c) right side positioning of filament.

The longitudinal view of composite yarns with different filament positioning is shown in Fig. 3. It can be clearly seen that the wrapping effect was better for the wrapped elastic composite yarn with filament positioning on the left (Fig. 3(a)) and right (Fig. 3(c)) sides than centre positioning (Fig. 3(b)).

**Fig. 4:**
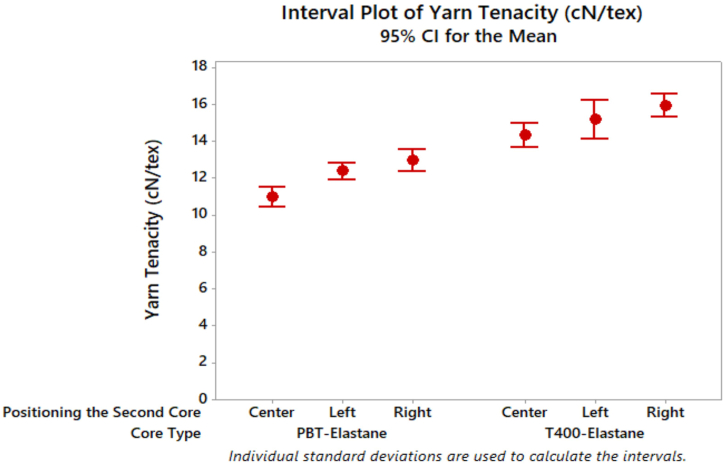
Tenacity results of composite yarn samples.

Upon analysis of the core type and positioning of the second core parameters, it was observed that the yarn tenacity values were lowest when PBT and elastane were fed together at the centre, as illustrated in Fig. 4. A review of the literature reveals that when the tenacity properties of dual-core spun yarns are examined, it is shown in similar studies that PBT-containing yarns have a lower tenacity compared to T400 yarns [27,29,45,50,51].

A change in the filament positioning from the centre to the right side resulted in a slight increase in tenacity. Both PBT and T400, when used as the second core component, demonstrated similar or higher tenacity compared to the centre filament positioning. The highest tenacity was achieved when T400 was used as the core component and wrapped with elastic composite yarn positioned on the right side. This result may be attributed to the twist direction; when twisted to the right, a "Z" twist is imparted. Since the filament core is added in the same direction as the twist, it is possible that it contributed to the improved tenacity. Similarly, when the wrapped elastic composite yarn had a PBT filament core component and was positioned from the right side, a similar result was observed. The type of filament core had a noticeable impact, with the T400 filament core component contributing to the strength of the yarn samples in all positioning types. This is likely due to the higher strength of the T400 filament compared to the PBT filament. Furthermore, the wrapped elastic composite yarns exhibited higher tenacity than the dual core-spun yarns, indicating the successful implementation of new feeding mechanisms. The results of the ANOVA in Table 3 confirmed that both the core type and the positioning of the second core significantly affected the tenacity of the yarn. However, the interaction (A*B) had no statistically significant effect on the tenacity of the yarn.

**Table 3:** ANOVA test results of composite yarn samples properties.

Dependent Variable	Independent Parameters		
	Core Type (A)	Positioning the Second Core (B)	A^a^B
Tenacity (cN/tex)	0.000^a^	0.000^a^	0.599
Breaking elongation (%)	0.000^a^	0.000^a^	0.000^a^
Unevenness (CVm%)	0.176	0.000^a^	0.001^a^
Hairiness (Uster H)	0.801	0.000^a^	0.871

a The mean difference is significant at the 0.05 level.

**Fig. 5:**
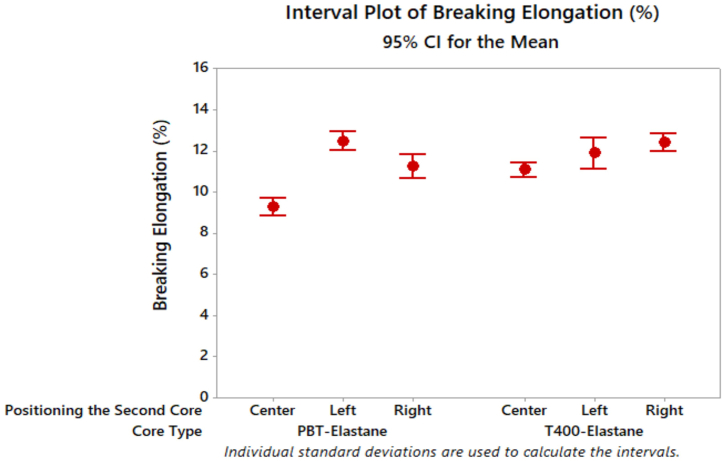
Breaking elongation results of composite yarn samples.

Tukey's test for tenacity values revealed no significant difference between the average yarn tenacity values for the right and left side positioning of the wrapped elastic composite yarns at a significance level of 0.05 (Table 4). However, the centre positioning of the filament was found to be statistically different from the others. It was observed that the wrapped elastic composite yarn with the filament on the right side exhibited the highest tenacity value. Fig. 5 presents the outcomes of the yarn breaking elongation test on distinct yarn samples.

**Table 4:** Tukey's multiple comparison test results of yarn properties in terms of positioning of second core parameter.

Tenacity (cN/tex)					
Positioning the Second Core	N	Mean	Grouping		
Right	20	14.4305	A		
Left	20	13.7525	A		
Center	20	12.6315		B	
Breaking elongation (%)					
Positioning the Second Core	N	Mean	Grouping		
Left	20	12.1720	A		
Right	20	11.8240	A		
Center	20	10.1645		B	
Unevenness (CVm%)					
Positioning the Second Core	N	Mean	Grouping		
Center	20	10.7840	A		
Left	20	10.6400		B	
Right	20	10.4755			C
Hairiness (Uster H)					
Positioning the Second Core	N	Mean	Grouping		
Center	20	6.5150	A		
Left	20	6.0930	A		
Right	20	5.0045		B

The elongation of the PBT content dual-core spun yarn (centre) was found to be slightly higher than that of T400 ones. These results regarding the elongation properties of PBT and T400-containing dual core spun yarns agree with previous studies [27,29,45,50,51]. The findings indicate that the elastic composite yarn with PBT as the core component on the left side exhibits the highest breaking elongation value. The breaking elongation of T400 core component composite yarns follows a trend from lowest to highest values with the filament positioned at the centre, left, and right. Furthermore, a slight increase in breaking elongation is observed in yarn samples with T400 filament as the second core component, moving from the centre to the right. In general, the second core component variable of T400 contributed more to the yarn elongation compared to PBT, with the exception of the breaking elongation of the yarn when combining the PBT filament from the left side as the second core component with elastane. Additionally, the core type, positioning of the second core, and interactions of A*B were found to significantly affect breaking elongation at a 5 % level (Table 3). The results of Tukey's multiple comparison test were consistent with those of the previous analysis, indicating that elastic composite yarns with the filament on the left side exhibited the highest breaking elongation. There was no statistically significant difference between the left and right side positioning of the filament (Table 4).

**Fig. 6:**
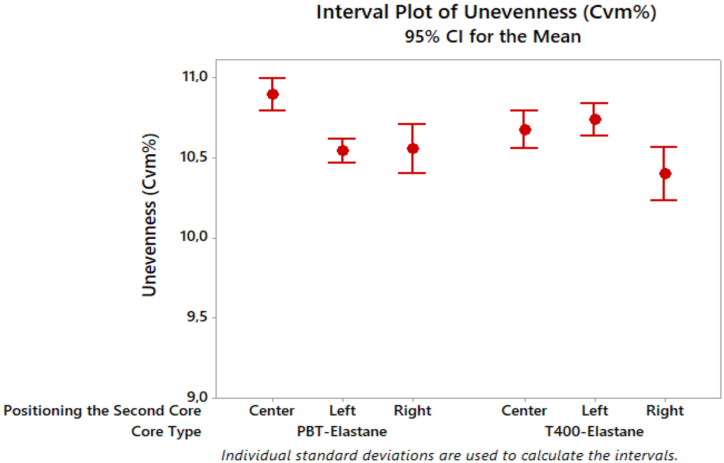
Unevenness results of composite yarn samples.

The results of the yarn unevenness indicate a similar coefficient of variation in mass (CVm%) for all yarn samples (Fig. 6). This similarity in unevenness can be attributed to the similar covering effect of one of the filament core components, either PBT or T400, together with the elastane core component. The wrapped elastic composite yarn with the T400 filament positioned on the right side (elastane at the centre) exhibited the least unevenness.

**Fig. 7:**
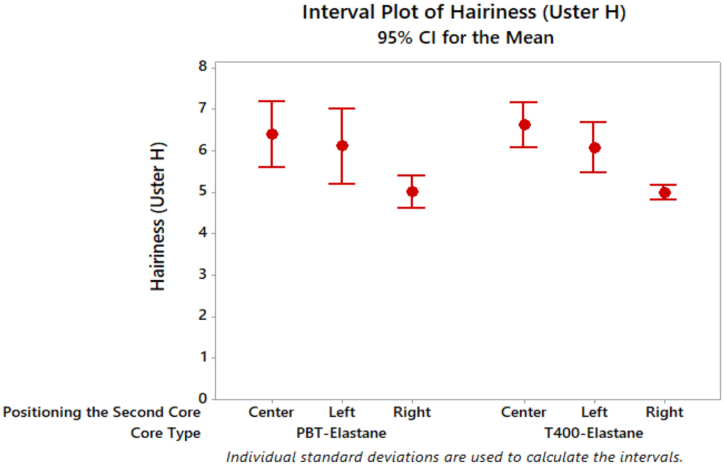
Hairiness results of composite yarn samples.

Table 3 indicates that the core type did not have a significant effect on yarn unevenness (p = 0.176), in contrast to the positioning of the second core. However, the interaction between core type and the positioning of the second core had a significant effect on yarn unevenness at a 5 % significance level. Further analysis using Tukey's multiple comparisons revealed that the subgroups were statistically different from each other (Table 4). Furthermore, the values were arranged in descending order, with the centre position representing the highest value, followed by the left and right positions (Center > Left > Right). Fig. 7 presents the results of the hairiness test on the yarn samples.

Fig. 7 illustrates that the wrapping of elastic composite yarns results in a reduction in hairiness when compared to the case where the filament is placed on the left side. The wrapping of the filament around the yarn surface serves to reduce the hairiness by capturing the fibre ends. With regard to the second core parameter, the positioning of the filament in the centre resulted in the highest hairiness values, while the positioning of the filament on the right side resulted in the lowest hairiness values for both PBT and T400 filaments. The examination of the effects of core type on yarn hairiness did not reveal any significant differences. The hairiness parameter is influenced by a number of factors, including elastane draw, filament draw, yarn count, linear density, sheath fibre raw materials and twist factor. It can be concluded that, provided that all other parameters remain constant, the use of any filament with the same linear density will result in a comparable effect on the hairiness of the yarn in this study. The results of the ANOVA for yarn hairiness demonstrated that the core type did not statistically significantly influence the outcome. However, the positioning of the second core parameter was found to have a significant effect on yarn hairiness (Table 3). Furthermore, it was observed that A*B interactions also had a statistically significant effect on yarn hairiness. Upon comparison of the subgroups of hairiness values, no statistical difference was found between the left and centre positioning of the filament. Furthermore, the yarns with the filament positioned on the right side exhibited the lowest hairiness value (Table 4).

### 4.2 Denim fabric properties

Table 5 presents the structural properties of the denim fabric. In order to evaluate the performance of the denim fabric, various properties were analysed, including the breaking load, tear force, elasticity and growth (after 30 min and 2 h relaxation time) in the weft direction. As the composite yarn samples are used as weft yarn in denim weaving production, all test results will be evaluated in the weft direction.

**Table 5:** Structural properties of denim fabrics.

Core Type	Positioning the Second Core	Warp Density (ends/cm)	Weft Density (picks/cm)	Dry Weight (g/m^2^)	Washed Weight (g/m^2^)
PBT + Elastane	Right	28	20	298	365
PBT + Elastane	Left	28	20	302	363
PBT + Elastane	Center	28	20	304	363
T400+Elastane	Right	28	20	306	368
T400+Elastane	Left	28	20	303	375
T400+Elastane	Center	28	20	306	375

### 4.3 Breaking load and tear force of denim fabrics

**Fig. 8:**
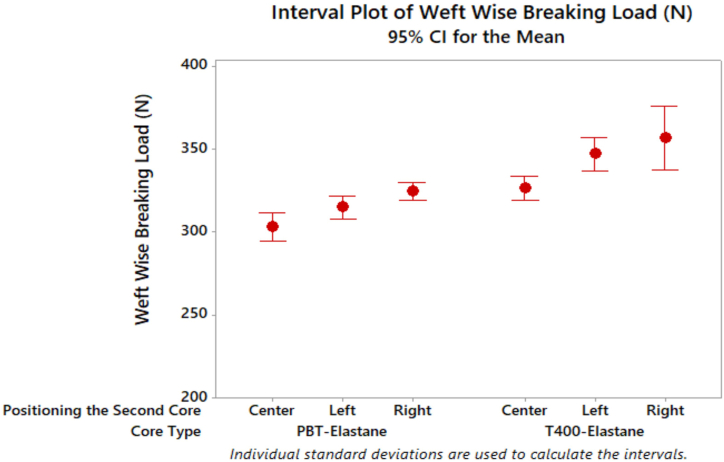
Weft wise breaking load results of denim fabrics.

Fig. 8 illustrates the breaking load of denim fabrics on a weft wise. Denim fabrics manufactured from dual core-spun yarn with PBT and elastane in the centre exhibited a lower breaking load compared to those manufactured from T400. However, the tenacity value of the dual core-spun yarn with T400 filament and elastane in the centre was higher than that of the PBT filament. This suggests that the higher tenacity value of the T400 dual core yarn contributes to the breaking load of the denim fabric.

The breaking load of denim fabrics increased in a direction from the centre to the right for both PBT and T400 core types. Denim fabrics produced with elastic composite yarns with filament positioning on the right side exhibited the highest breaking load. This is attributed to the favourable winding effect resulting from the filament being fed from the right side, which contributes to higher yarn tensile properties and denim fabric breaking load. Denim fabrics with T400 filament cores exhibited a higher breaking load in the weft direction than those with PBT filaments, which can be attributed to the higher strength of the T400 filament. Denim fabrics with wrapped elastic composite yarns demonstrated a higher breaking load in general. The results of the ANOVA test, presented in Table 6, indicate that, in contrast to the interaction A*B (p = 0.558), both the core type and the positioning of the second core significantly influence the breaking load of denim fabrics in the weft direction.

**Table 6:** ANOVA test results of denim fabrics properties.

Dependent Variable	Core Type (A)	Positioning the Second Core (B)	A^a^B
Weft wise breaking load (N)	0.000^a^	0.000^a^	0.558
Weft wise tear force (cN)	0.000^a^	0.000^a^	0.084
Elasticity (%)	0.000^a^	0.000^a^	0.001^a^
Growth after 30 min (%)	0.000^a^	0.000^a^	0.003^a^
Growth after 2 h (%)	0.000^a^	0.022^a^	0.641

a The mean difference is significant at the 0.05 level.

The results of the Tukey test in Table 7 indicate that there is no statistically significant difference between the left and right side positioning of the filament denim fabric in terms of weft wise breaking load values. However, it is noteworthy that denim fabrics produced from dual core spun yarns have the lowest breaking load values.

**Table 7:** Tukey's multiple comparison test results of denim fabric properties in terms of positioning of second core parameter.

Weft Wise Breaking Load (N)					
Positioning the Second Core	N	Mean	Grouping		
Right	20	340,781	A		
Left	20	330,974	A		
Center	20	314,793		B	
Weft Wise Tear Force (cN)					
Positioning the Second Core	N	Mean	Grouping		
Left	20	3736,240	A		
Right	20	3646,650	A		
Center	20	3445,130		B	
Elasticity (%)					
Positioning the Second Core	N	Mean	Grouping		
Center	20	55.060	A		
Left	20	54.620	A		
Right	20	52.820		B	
Growth after 30 min (%)					
Positioning the Second Core	N	Mean	Grouping		
Center	20	10.621	A		
Left	20	9.820		B	
Right	20	9.640		B	
Growth after 2 h (%)					
Positioning the Second Core	N	Mean	Grouping		
Center	20	6.220	A		
Left	20	6.100	A	B	
Right	20	5.840		B	

**Fig. 9:**
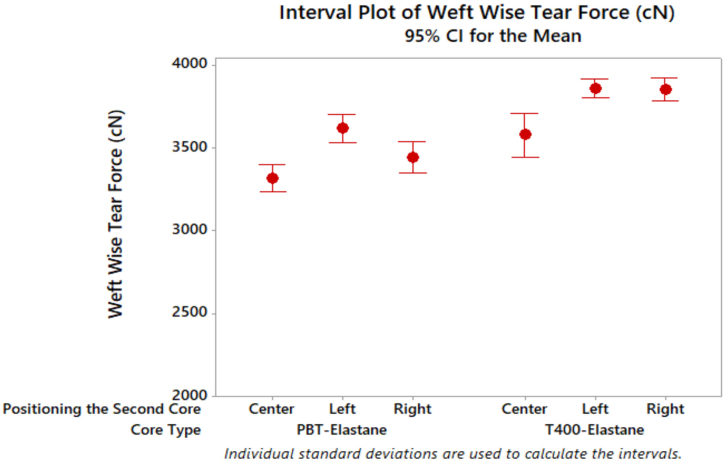
Weft wise tear force results of denim fabrics.

Weft wise tear force test results of denim fabrics are given in Fig. 9. As observed in the literature, the tear strength of denim fabric produced from dual core spun yarns with T400 content fed in the centre is higher than that of fabric produced from PBT content [23]. Furthermore, denim fabrics with T400 core filament demonstrated higher tear force than those produced from PBT fabrics. The positioning of the second core had a significant impact on the weft-wise tear force results of denim fabric, particularly for the right side filament positioning of the T400 core component.

Furthermore, denim fabrics made with wrapped elastic composite yarns exhibited a higher tear force in the weft direction compared to denim fabrics made with dual core yarns. In this context, the presence of filaments had a greater effect, attributed to the filament twisting of the yarn when fed from both the right and left side. Conversely, as illustrated in Table 6, the type of core and the positioning of the second core had a significant impact on the weft-wise tear force, while the A*B interaction (p = 0.084) did not have a significant effect. The highest weft direction tear force was observed in denim fabrics with the filament yarn positioned on the left side. Furthermore, no significant difference was identified between the weft-direction tear forces of denim fabrics produced with wrapped elastic composite yarns positioned on the left and right sides (Table 7).

### 4.4 Elasticity and growth properties of denim fabrics

**Fig. 10:**
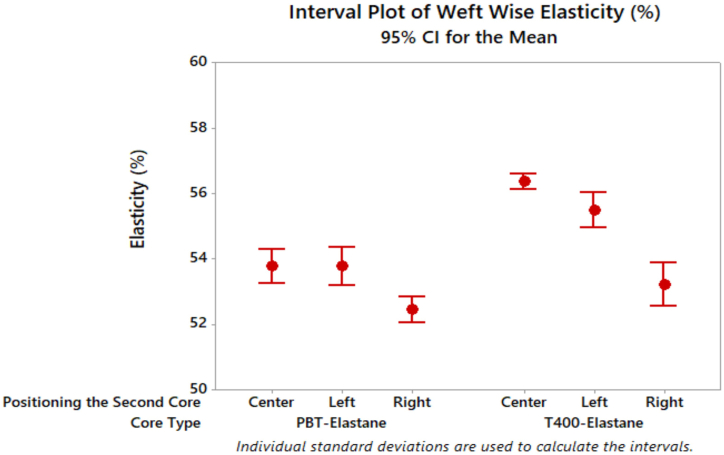
Elasticity results of denim fabrics.

Upon examination of the results of the elasticity test on denim fabrics (Fig. 10), the lowest values were obtained from denim fabrics produced using PBT filament of wrapped elastic composite yarn from the left side feeding.

**Fig. 11:**
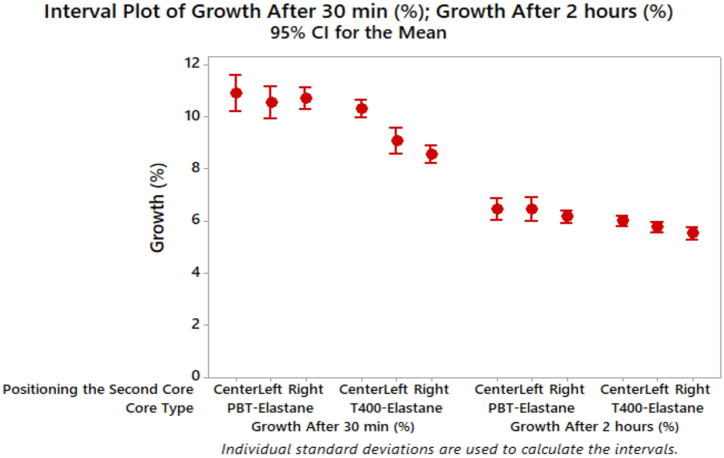
Growth results of denim fabrics.

The results demonstrate that denim fabrics with T400 filament exhibit higher elasticity, which can be attributed to the fabric's comfortable fit when worn. Among the denim fabrics tested, the fabric with dual core-spun yarn (T400+elastane) exhibited the highest elasticity value, which is similar to the results reported in literature [37]. It is noteworthy that the positioning of the filament on the left or right side did not affect the elasticity properties for both core types (PBT and T400), as similar elasticity behaviour was observed. Furthermore, fabrics with dual core-spun yarns using both PBT and T400 filaments demonstrated higher elasticity compared to fabrics with wrapped elastic composite materials. The statistical analysis verified that the core type, positioning of the second core, and the interaction between these parameters exerted a statistically significant influence on the elasticity of the denim fabrics (Table 6). Moreover, the results of Tukey's test (Table 7) indicated that the mean elasticity values for the left and center positioning of the filament were not significantly different, while the elasticity of denim fabric containing filament positioned on the right side differed from the others. Fig. 11 displays the growth results of denim fabrics after 30 min and 2 h of relaxation time.

Denim fabrics incorporating T400 filament exhibited lower growth indices and higher elastic recovery after 30 min of relaxation time. The growth of denim fabrics decreased from the centre to the right side positioning of the filament (Centre > Left > Right), with the exception of the left side positioning of T400 filament, which deviated from this trend. All independent parameters and their interaction had a statistically significant effect on growth after 30 min of relaxation time (Table 6). With regard to the growth of denim fabrics following a 2-h period of relaxation, a decreasing trend emerged with respect to the positioning of the second core (Center > Right > Left). In a manner consistent with the observations made following a period of 30 min, T400 filament exhibited superior recovery capabilities compared to PBT filament. It was concluded by Ute et al. that the growth property of denim fabric obtained from PBT-containing dual core spun yarn was lower than that obtained for T400, which agrees with the findings of this study [37].

Denim fabrics with wrapped elastic composite yarns demonstrated a reduction in growth following a 2-h relaxation period. The effects of both core type and the positioning of the second core on fabric growth following a 2-h relaxation period were statistically significant, with the exception of the interactions between these parameters. The growth of denim fabrics indicated that denim fabrics with wrapped elastic composite yarns contributed to a decrease in growth. As illustrated in Table 7, the growth of denim fabric with right-side positioning filament was the lowest after both 30 min and 2 h of relaxation time. Additionally, the growth of denim fabric with centre-positioning filament differed from that of the left and right ones after 30 min. Furthermore, it was concluded that the growth of denim fabric with right-side positioning filament was the lowest after 2 h of relaxation time and was not different from that of the left ones.

## 5 Conclusion

In this study, the effect of both core type and positioning of the second core parameters were investigated on yarn and denim fabric properties. The results are summarized as follows.

• Wrapped elastic composite yarns produced using a "W-grooved" roller demonstrated enhanced properties in terms of tenacity, breaking elongation, unevenness, and hairiness. Yarns with T400 filament also exhibited elevated tenacity. The positioning of the second core component had a pronounced impact on these properties. In general, wrapped elastic composite yarns with filament on the right and left sides exhibited superior properties compared to those in the centre. Furthermore, yarns with filament on the right side exhibited the lowest values for unevenness and hairiness.
• The T400 filament demonstrated superior performance in terms of weft-wise breaking load and tear force in denim fabrics when compared to the PBT filament. The incorporation of wrapped elastic composite yarns as weft yarns led to notable enhancements in fabric properties, including weft-wise breaking load, tear force, elasticity, and lower growth. The core type and positioning of the second core exhibited a statistically significant impact on all denim fabric properties examined. The weft-wise breaking load and tear force values of denim fabrics with right-side positioning filament were not found to be significantly different from those with left-side positioning filament.

In this study, an alternative method was explored to improve yarn and fabric properties, using a "W-grooved" roller for composite yarn production. As a result of this study, it was found that changing filament positioning with the "W-grooved" roller resulted in enhanced yarn and denim fabric performance.

The apparatus developed in this study was designed to enhance the functionality of the product, thereby improving its quality and performance characteristics, as mentioned before. The results of this study demonstrate that the objective has been achieved. It can be observed that the apparatus developed and patented can be used to produce value-added products, which will be advantageous for the producer. Consequently, it is postulated that the utilisation of such apparatus, which may be perceived as an additional cost item, may ultimately confer advantages upon yarn manufacturers in both the development of product range and profitability.

## CRediT authorship contribution statement

**Osman Babaarslan:** Project administration, Formal analysis, Data curation. **Esin Sarıoğlu:** Writing – review & editing, Writing – original draft, Investigation. **Onur Duru:** Resources, Methodology.

## Declaration of competing interest

The authors declare that they have no known competing financial interests or personal relationships that could have appeared to influence the work reported in this paper.

## References

[1] Sawhney A.P.S., Robert K.Q., Ruppenicker G.F. (1989). Device for producing staple-core/cotton-wrap ring spun yarns. Textil. Res. J..

[2] Sawhney A.P.S., Ruppenicker G.F., Kimmel L.B., Robert K.Q. (1992). Comparison of filament-core spun yarns produced by new and conventional methods. Textil. Res. J..

[3] Matsumoto Y., Toriumi K., Tsuchiya I. (1992). Properties of double-core twin spun silk yarns and fabrics. Textil. Res. J..

[4] Wu W.Y., Lee J.Y. (1995). Effects of spread width on the structure, properties and production of a composite yarn. Textil. Res. J..

[5] Su C., Lee J., Wu W. (1998). Effect of oil composition on physical properties and spread width of polyester multifilament for spinning a composite yarn. J. Chin. Inst. Eng..

[6] Babaarslan O. (2001). Method of producing a polyester/viscose core-spun yarn containing spandex using a modified ring spinning frame. Textil. Res. J..

[7] Pourahmad A., Johari M.S. (2009). Production of core-spun yarn by the three-strand modified method. J. Text. Inst..

[8] Gharahaghaji A.A., Zargar E.N., Ghane M., Hossaini A. (2010). Cluster-spun yarn-a new concept in composite yarn spinning. Textil. Res. J..

[9] Ralph B., Tharpe J.R., John L., Allen J.R., Little F.A., Reuben E.H. (2012).

[10] Naeem M.A., Yu W., Zheng Y.H., He Y. (2014). Structure and spinning of composite yarn based on the multifilament spreading method using a modified ring frame. Textil. Res. J..

[11] Kim M., Jeon B.S. (2014). New composite yarn with staple fiber and filament controlling the delivery speed ratio. Fibers Polym..

[12] Matsumoto M., Matsumoto Y., Kanai H., Wakako L., Fukusima K. (2014). Construction of twin staple-core spun yarn with two points of yarn formation in one twisting process. Textil. Res. J..

[13] Naeem M.A., Akankwasa N.T., Leroy A., Siddiqui Q., Ahmad A. (2018). A study of novel multifilament spreading and feeding method, to produce filament wrapped-staple core composite yarn using modified ring frame. J. Text. Inst..

[14] Song J., Su X., Liu X. (2019). Study on shape retention properties of filament/staple fiber composite yarns and fabrics. Int. J. Cloth. Sci. Technol..

[15] Jiang W., Guo M., Gao W. (2023). Research on a novel sheath core-wrap staple yarn based on the ring spinning frame with special-shaped roller. Textil. Res. J..

[16] Çelikkan Aydoğdu S.H., Yılmaz D. (2020). Effect of yarn fineness and core/sheath fibre types on the physical properties of dual-core yarns and fabrics. Cellul. Chem. Technol..

[17] Erbil Y., Islam R., Babaarslan O., Sırlıbaş S. (2020). Effect of structural changes on the cotton composite yarn properties. J. Nat. Fibers.

[18] Su X., Liu X. (2020). Research on performance of twin-core spun yarn and fabric. Int. J. Cloth. Sci. Technol..

[19] Telli A., Daşan Y., Babaarslan O., Karaduman S. (2017). Usage of core and dual-core yarns containing tungsten for electromagnetic shielding. Adv Res Text Eng.

[20] Bedez Ute T., Kadoğlu H. (2018). The effect of core material parameters on the mechanical properties of double core and single core spun yarns. IOP Conf. Ser. Mater. Sci. Eng..

[21] Turksoy H., Ertek Avcı M., Yıldırım N. (2020). The effects of production parameters on the physical properties of dual-core slub yarns. Tekstil ve Konfeksiyon.

[22] Hua T., Wong N.S., Tang W.M. (2017). Study on properties of elastic core-spun yarns containing a mix of spandex and PET/PTT bi-component filament as core. Textil. Res. J..

[23] Babaarslan O., Shadid M.A., Doğan F.B. (2021). Comparative analysis of cotton covered elastomeric hybrid yarns and denim fabric properties. Journal of Engineered Fibers and Fabrics.

[24] Chhatpuriya A., Maity S., Kumar Sinha S. (2022). Stress relaxation and elastic recovery behaviour of dual core stretchable ring spun yarn. Journal of Textile Engineering & Fashion Technology.

[25] Babaarslan O., Sarıoğlu E., Ertek Avcı M. (2019). A comparative study on performance characteristics of multicomponent core-spun yarns containing Cotton/PET/elastane. J. Textil. Inst..

[26] Turksoy H.G., Yıldırım N. (2018). Effect of process variables on the properties of dual-core yarns containing wool/elastane. Industria Textila.

[27] Vuruşkan D. (2019). Effects of sheath fiber and core material on dual core-spun yarns quality properties. J. Textil. Eng..

[28] Çelikkan Aydoğdu S.H., Yılmaz D. (2019). Analyzing some of the dual-core yarn spinning parameters on yarn and various fabric properties. Tekstil ve Konfeksiyon.

[29] Kurban N.S., Babaarslan O. (2022). The effect of fiber types used as core/wrapper fibers in multi-component yarns on the stretch properties of denim fabrics. J. Nat. Fibers.

[30] Chen C., Cao J., Qin X., Ren J., Kong D., Han Q., Meng X., Yu J. (2022). A novel concept to produce submicron-cotton/polyester composite core-spun yarn via modified apparatus. J. Nat. Fibers.

[31] Okyay G., Demiryürek O., Avci M.E., Bilgiç H. (2023). Development and characterization of hemp-containing hybrid yarns for clothing. Cellul. Chem. Technol..

[32] Elrys S.M.M.E., Habiby F.F.E., Elkhalek R.A., El-Deeb A., El-Hossiny A.M. (2021). Investigation into the effects of yarn structure and yarn count on different types of core-spun yarns. Textil. Res. J..

[33] Elrys S.M.M.E., El-Habiby F.F., Eldeeb A.S., El-Hossiny A.M., Abd Elkhalek R. (2023). Comfort properties of knitted fabrics produced from dual-core and tri-core spun yarns. Textil. Res. J..

[34] Fan W., Zhang Y., Sun Y., Wang S., Zhang C., Yu X., Wang W., Dong K. (2023). Durable antibacterial and temperature regulated core-spun yarns for textile health and comfort applications. Chem. Eng. J..

[35] Ertaş O.G.B., Zervent Ünal B., Çelik N. (2016). Analyzing the effect of the elastane-containing dual-core weft yarn density on the denim fabric performance properties. J. Textil. Inst..

[36] Babaarslan O., Sarioğlu E., Çelik H.İ., Ertek Avcı M., Singh Mukesh Kumar (2018). Engineered Fabrics.

[37] Bedez Ute T. (2019). Analysis of mechanical and dimensional properties of the denim fabrics produced with double-core and core-spun weft yarns with different weft densities. J. Textil. Inst..

[38] Yılmaz D., Çelikkan Aydoğdu S.H. (2019). Analyzing some of the dual-core yarn spinning parameters on yarn and various fabric properties. Textile and Apparel.

[39] El-Tantawy S., Sabry M., Bakry M. (2017). The effect of different weft yarn production technique on the pilling property of jeans fabrics. International Design Journal.

[40] Daşan Y., Babaarslan O. (2020). Stretch and physical properties of weft stretch denim fabrics containing elastane and filament yarn. Journal of Textile Science & Fashion Technology.

[41] Erbil Y., Babaarslan O., Islam R., Sırlıbaş S. (2022). Performance of core & dual-core cotton yarn structures on denim fabrics. J. Nat. Fibers.

[42] Rahim Md A., Rahman S., Uddin A.J. (2023). Low-bagging (growth) stretch denim yarn production by spinning optimization of cotton-wrapped dual-core elastane and T400 multifilament. Heliyon.

[43] Irfan M., Qadir M.B., Afzal A., Shaker K., Salman S.M., Majeed N., Indrie L., Albu A. (2023). Investigating the effect of different filaments and yarn structures on mechanical and physical properties of dual-core elastane composite yarns. Heliyon.

[44] Babaarslan O., Shahid M.A., Doğan F.B. (2023). Design of hybrid yarn with the combination of fiber and filaments and its effect on the denim fabric performance. Fibres Text. East. Eur..

[45] Rahim M.A., Rahman S., Uddin A.J. (2023). Low-bagging (growth) stretch denim yarn production by spinning optimization of cotton-wrapped dual-core elastane and T400 multifilament. Heliyon.

[46] Öztürk Ö. (September 18, 2018).

[47] Nergis B. (2017). An overview of hybrid ring spinning methods. Current Trends in Fashion Technology & Textile Engineering.

[48] Liu W., Yu Y.P., He J.H., Wang S.Y. (2007). Effect of tension compensator on sirofil yarn properties. Textil. Res. J..

[49] Xia Z., Guo Q., Ye W., Chen J., Feng S., Ding C. (2018). Comparative study of fiber trapping by filaments in conventional and diagonal sirofil systems. Textil. Res. J..

[50] Habib A., Olgun Y., Babaarslan O. (2024). Development of dual-core spun yarn using different filaments as a core and its impact on denim fabric properties. Textile & Leather Review.

[51] Habib A., Mamun M.A.A., Babaarslan O. (2024). Development of sustainable dual core-spun yarns using several filaments and recycled cotton sourced from pre-consumer fabric waste. Heliyon.

